# Are There Physiological Affinities in the Blood of Each Breed of Animals

**Published:** 1883-04

**Authors:** A. S. Heath

**Affiliations:** Professor of Bovine, Ovine and Suine Pathology, Columbia College of Comparative Medicine, &c.


### Art. XI.—ARE THERE PHYSIOLOGICAL AFFINITIES IN THE BLOOD OF EACH BREED OF ANIMALS? No. 11.

BY A. S. HEATH, M.D.,

*Professor of Bovine, Ovine and Suine Pathology, Columbia College of Comparalive Medicine, &c.*

THE subject of blood affinities of our various breeds of domestic animals for each other of the particular strains, is full of significance. When we come to learn from whence our best mixed blooded animals were derived, and perceive the peculiar improvements in our grades by using pure-bred males in breeding, specially for the several distinctive industries, then we will be able to more fully appreciate the value of blood affinities of each breed for its own kind. To make the matter plainer, let us see from whence we originally derived our domestic animals in the early settlement of the country. And first, let us see where our cows came from.

The early settlers of Maine from Suffolk, England, brought with them the Suffolk Dun cattle. The characteristics of these

Dun cattle, in color and other respects, are still noticed in the State of Maine. The blood of this breed is mixed and greatly diluted, because no recent infusion of the same blood has taken place. Now, if the people of Maine should desire to revive the Suffolk Dun breed of cattle where they still have grades of that breed, and if they could secure pure-bred Suffolk Dun males from England, they could accumulate the characteristics of these animals by accumulating the blood of the Dun breed. This rule will apply to fortifying the blood of any grades with the real, noble, original blood, by enriching it from its source, or fountain head. This enriching or concentrating the blood of special breeds brings out the original qualities—color, form, size, aptitude for butter, cheese, quantity of milk, for beef, or for labor.

In 1640 Massachusetts colony told 21,500 people. These came from every part of England. They were intelligent, practical and sturdy. Among them were the best farmers, who brought and imported the best cattle England possessed. The hardy, active, rich dark-red Devons were prominent in the even small herds of that day. The blood characteristics of these beautiful animals are everywhere traced in the grades or mixed common stock of that State and elsewhere. Select of these grades cows that show some Devon blood, and place in such herds pure-bred Devon bulls, and soon there will cumulate Devon blood and Devon characteristics. The rich dark-red color will appear, the yellow skin, the compact form, the quick step, the rich milk, in a word, the Devon breed will return with each infusion of pure blood. But this will not occur with the accumulation of the purest blood of any other breed crossed upon these Devon grades. The blood affinities, therefore, of breeds for each other does exist.

Besides the breeds of cattle above referred to, there came early to America the Long Horns; the red Glamorgans; the hardy Pembrokes; the Angleseys; the polled Norfolks; the solid Herefords; the Sussex; old Durham; the old Jerseys mixed with the Brittany and Norman stock; the grand old Yorkshires, always large milkers; the dark-brown and brindle cattle of Wales; the Holderness; the black and white Dutch; the Scotch breeds, including the polled Galloways; the Irish breeds; the Swiss; the German breeds, the glory of the modern dairies of Germany, and others, now forming foundations for grades of surpassing excellence. The breeds of our ancestors were numerous and well bred. And though our dairies are composed of these mixtures, and although 40 per cent. of our cows are worthless for any product, yet the other 60 per cent. of cows possess rich, though divergent and diverse blood affinities, yet, when bred *up* by pure males of special capabilities for the different products—milk, butter, cheese, beef, labor—they produce the finest grades of any country. American common or mixed cattle possess good blood, with vigorous breed affinities, and thus make superlative *grades*, breeding *up* profitably with the infusion of the pure blood of those breeds whose blood predominates in them. Thus, a cow having more blood of the Shorthorn breed than of any other, has more active blood affinities for the pure Shorthorn blood than for the blood of any other breed. This holds good with the Ayrshire cow, the Dutch cow, the Jersey, the Cotentine, the Charolais, the Allgaur, the Guernsey, the Swiss, the cow of whatsoever breed, provided that the blood of her breed predominates when again mixed with pure blood of the same breed. Even if there is no predominence of the blood of any particular breed, yet the infusion of a special kind of blood, and this renewed each succeeding generation, cumulates breed affinities of blood, which show all the characteristics of that particular breed strongly marked in the offspring of these unions.

The Hereford, the Angus, the Limousine, the Shorthorn, all have strongly marked beef characteristics; so, indeed, the Normandy, the Jersey, the Guernsey, the Swiss, have as well marked butter characteristics; the Ayrshire, the Limberger, the Angler, the Allgaur, have prominent cheese producing powers; while the Holstein, the Flamande and the Yorkshire have large milk yielding possiblities. Though the blood of the best of these breeds may affiliate and produce fine animals, yet, the blood of the beef breeds mixed with the animals of these peculiar characteristics produce better and more profitable results. And equally is this true of the breeds of cattle for the other special products. Then there are not only blood affinities of breeds, but also blood affinities of the different breeds of similar characteristics. Butter breeds affiliate most profitably with butter breeds; cheese breeds with cheese breeds. In other words, animals predisposed to certain characteristics have stronger blood affinities towards other animals and breeds with like proclivities. Blood stamps its characteristics strongest and best where these characteristics are indelibly fixed and inbred.

Breed affinities and product affinities are two very different matters; yet, both kinds of blood affinities often unite in one breed. *Beef animals* of different breeds, or *butter animals* of diverse breeds, have strong blood affinities for these powers which produce these products in these breeds. But stronger still are the blood affinities—the physiological aptitudes—the vital activities—of breeds with breeds of the same consanguinity, and predisposed to eliminate a greater quantity of a certain product from the same given quantity and quality of food.

The commingling of the blood of *butter breeds* produces large products of butter; but the reunion of the blood of Jersey with Jersey, or Guernsey with Guernsey, is more consonant and produces more stable and uniform characteristics.

By breeding and feeding a herd of the same blood for a long time for any particular product, say beef, a sagacious breeder persistently selects animals of the best form for beef; animals having good digestion and surpassing powers of food assimilation for beef production; those that mature early; those that are hardy; those that have vigorous constitutions; always breeding together these animals whose physiological characteristics have changed and fixed in the production of beef of the largest amount and of the best quality, in the shortest time, and with the least expenditure of food. In this way, and by such methods, any product may be secured in any breed of cattle. But it is a great economy to select the breed for a special product whose proclivities, tendencies and physiological aptitudes bespeak satisfactory results. For instance, a Shorthorn, so long bred for beef, is not soon nor cheaply converted into a butter or cheese producer. The same rule will apply even more strongly to the other beef breeds; for in the

*make up* of the Shorthorn breed valuable strains of blood of a milk breed, or breeds, were infused.

It is not asserted that breeds have but one excellence; but it is certain that no animal, or no breed of animals, possessed two superlative qualities at the same time. A breed raised for a particular product, and possessing the powers to produce that product superlatively in all respects, may have other excellent capabilities, but never in so marked and transcendant degree. As a general rule, the Shorthorn, the Devons, the Hereford, the Angus, the West Highland breeds, are beef producers. Some of these are good for labor, some for milk or butter, but none of them are equally great for two or more products. Some of the beef breeds make good working oxen. The Charolais, Limonsine and Devons are beautiful animals, good oxen, and, when well fed, make beef that even the English prefer to that of the Shorthorn, the Hereford, or the Angus.

This does not in any way detract from those grand beef breeds, for even the remote grades of these breeds surprisingly show beef qualities in form and substance.

Then we are correct in asserting that there are physiological affinities of blood of the several breeds of cattle; such affinities being strongest in the blood of each particular breed for each other; and relatively strong for a product where the aptitude for a special product exists in both breeds of animals crossed to promote or secure one and the same product in a superlative degree.

Then, successful breeding requires a knowledge of the relative blood affinities; the physiological aptitudes of breeds for a special object; the tendencies of different breeds towards certain products; the correllation of part to part and product to product; also a thorough understanding of food values, and the value of sanitary measure in the breeding and rearing of our domestic animals. It is through the teachings of Veterinary Science that such knowledge must be sought and obtained. When the thoroughly educated Veterinary Surgeon shall be consulted on the subjects of breeding, management, and treatment of our domesticated animals, then the farmer will appreciate the value in economy, humanity and satisfaction, the important services of the members of so learned a profession as that of Comparative Medicine and Surgery.

